# The impact of population ageing on end-of-life care in Scotland: projections of place of death and recommendations for future service provision

**DOI:** 10.1186/s12904-019-0490-x

**Published:** 2019-12-12

**Authors:** Anne M. Finucane, Anna E. Bone, Catherine J. Evans, Barbara Gomes, Richard Meade, Irene J. Higginson, Scott A. Murray

**Affiliations:** 1 Marie Curie Hospice Edinburgh, 45 Frogston Road West, Edinburgh, Scotland, UK; 2 Usher Institute, University of Edinburgh, Edinburgh, Scotland, UK; 3 Cicely Saunders Institute of Palliative Care, Policy and Rehabilitation, King’s College London, Bessemer Road, Denmark Hill, London, SE5 9PJ UK; 4 Faculty of Medicine, University of Coimbra, Coimbra, Portugal; 5 Policy and Public Affairs for Scotland, Marie Curie, Edinburgh, Scotland, UK; 6 Usher Institute, University of Edinburgh, Edinburgh, Scotland, UK

**Keywords:** Forecasts, Projections, Frailty, Palliative care, Place of death, Care homes, Nursing homes

## Abstract

**Background:**

Global annual deaths are rising. It is essential to examine where future deaths may occur to facilitate decisions regarding future service provision and resource allocation.

**Aims:**

To project where people will die from 2017 to 2040 in an ageing country with advanced integrated palliative care, and to prioritise recommendations based on these trends.

**Methods:**

Population-based trend analysis of place of death for people that died in Scotland (2004–2016) and projections using simple linear modelling (2017–2040); Transparent Expert Consultation to prioritise recommendations in response to projections.

**Results:**

Deaths are projected to increase by 15.9% from 56,728 in 2016 (32.8% aged 85+ years) to 65,757 deaths in 2040 (45% aged 85+ years). Between 2004 and 2016, proportions of home and care home deaths increased (19.8–23.4% and 14.5–18.8%), while the proportion of hospital deaths declined (58.0–50.1%). If current trends continue, the numbers of deaths at home and in care homes will increase, and two-thirds will die outside hospital by 2040. To sustain current trends, priorities include: 1) to increase and upskill a community health and social care workforce through education, training and valuing of care work; 2) to build community care capacity through informal carer support and community engagement; 3) to stimulate a realistic public debate on death, dying and sustainable funding.

**Conclusion:**

To sustain current trends, health and social care provision in the community needs to grow to support nearly 60% more people at the end-of-life by 2040; otherwise hospital deaths will increase.

## Background

Worldwide deaths are projected to rise from 55 million in 2016 to 75 million by 2040 [1]. Non-communicable diseases accounted for 72% of global deaths in 2016, and may account for 81% of deaths by 2040 [1]. Globally the proportion of people aged 80 or over increased by 77% between 2000 and 2015 [2]. These demographic shifts require care systems to adapt to address the concerns of the growing older population.

In Scotland, with a population of 5.4 million, deaths increased moderately over the last decade from 55,986 deaths in 2007 to 57,883 in 2017 [3]. Causes of death are changing, with more people dying from dementia and Alzheimer’s (4.6% in 2006 to 11.3% in 2017), and steady decreases in deaths from cerebrovascular or ischaemic heart disease (from 27.2% in 2006 to 18.4% in 2017) [3]. Deaths from cancer have remained stable (27.4% in 2006 to 28% in 2017). The average age at death in 2017 was 77 years, an increase from 75 years in 2006 [3]. People aged 85 and over accounted for one-third of all deaths in 2017; this is projected to rise to 45% by 2040 [3, 4].

An understanding of where people may die in the future is essential to facilitate planning and optimise resource allocation. It can act as a baseline measure to evaluate progress in shifting resources across care settings. Given that many people express a preference to die at home if circumstances allow [5], death in a community setting may act as a proxy indicator of whether preference was met.

Place of death trends differ considerably by country and may relate to the degree of palliative care integration within the wider health system. Trends in Scotland emerge within a context where specialist palliative care services are at a stage of advanced integration into mainstream service provision [6]. Comprehensive provision of all types of palliative care is offered by multiple providers and there is a broad awareness of palliative care on the part of health professionals, local communities, policy-makers and society. In Scotland, and across the UK, the integration of health and social care is progressing, aiming to facilitate greater co-ordination between health and social services in coming decades.

Countrywide studies have projected trends in place of death for England and Wales [7], Portugal [8], Germany [9] and Belgium [10]. Data from England and Wales project a 25% increase in the number of annual deaths by 2040; if current trends continue, more people will die at home and in care homes, with care home becoming the most common place of death by 2040 [7]. In Germany, home deaths and nursing home deaths are expected to increase [9], and in Belgium deaths in care homes are projected to rise [10].

However, in Portugal, a country with a scarcity of palliative care services and little integration, projections show that if current trends continue, hospital deaths will account for nearly three-quarters of all deaths by 2030 [8]. In countries with ageing populations where palliative care is localised and less well integrated into the broader system, the development of more integrated models of palliative care are required as a first step before significant shifts from death in hospital to community settings can be realised [8].

We aim to i) project where people in Scotland will die up to 2040 and ii) identify expert recommendations for future care provision based on the projected data. Our findings will inform decision-making regarding resource allocation, service commissioning and service innovation for end-of-life care in Scotland.

## Methods

### Study design

Population-based trend analysis and projections using simple linear modelling to project place of death in Scotland for each year from 2017 to 2040, building on the methods of Gomes and Higginson [11] and Bone et al. [7], followed by a Transparent Expert Consultation [12] to develop recommendations for service delivery to meet projected future needs.

### Data sources

We obtained routinely collected death registration data 2004–2016 by age and gender from the National Records of Scotland (NRS) [3]. We categorised place of death according to NRS reporting standards, with the exception of hospice deaths, which were separated from the ‘care home’ category by the NRS at our request (Additional file 1). Place of death categories included ‘own home’ (non-institution and person’s usual residence), ‘care home’ (includes nursing homes and residential homes without nurses), ‘NHS hospital’, ‘hospice’ and ‘other’ (e.g. road, shop, prison, school).

We accessed official projected future deaths for the population of Scotland (2017 to 2040) by age and gender from the Office of National Statistics (ONS) [4]. We used the ONS 2016-based national principal population projections [4]. Projected deaths were grouped into seven age categories (0–4 years, 5–14, 15–44, 45–64, 65–74, 75–84, 85 and over) and by gender.

### Data analysis

We first described the number and proportion of people dying in each age and gender group observed between 2004 and 2016, and the projected deaths by age and gender up to 2040. We calculated the proportion of people in each age and gender group who died in each care setting (2004–2016). We applied estimated age and gender-specific proportions of deaths by place of death to the projected deaths in each age and gender stratum for each year up to 2040 to estimate future place of death, based on an established methodology [7, 11, 13]. We modelled four scenarios. Scenarios 1–3 were derived from the latest England and Wales projections and allow comparability [7]. We added an additional scenario (Scenario 4) to allow us to project trends if primary and social care resource is limited to current levels and community deaths do not increase beyond 2016 levels.

- **Scenario 1** assumed no change in the age and gender specific proportions of deaths observed in 2016 in each place of death.
- **Scenario 2** assumed that the mean yearly change in age and gender specific proportions of deaths in each place of death that occurred between 2004 and 2016 continues.
- **Scenario 3** assumed that the mean yearly change in age and gender specific proportions of deaths in each place of death that occurred between 2004 and 2016 continues, but that care home deaths do not increase above the number observed in 2016, with any additional deaths instead occurring in hospital.
- **Scenario 4** assumed scenario 3 above, but that home deaths as well as care home deaths do not increase above their absolute numbers in 2016.

### Expert consultation

#### Participants

The study team identified experts in palliative care, primary care and social care with representation from commissioners, service providers, government, researchers and professionals from charities. Eligible participants received an email invitation to the consultation. Those who agreed to take part received a pre-workshop briefing pack and a participant consent form in advance, which they signed prior to the consultation.

#### Design

We used an abbreviated MORECare Transparent Expert Consultation approach consisting of a modified nominal group technique [12]. All data was collected during one half-day consultation event. At this event, we first presented to all participants (*n* = 27) place of death projections in Scotland from 2017 to 2040 based on the four scenarios described. Participants were then allocated to one of three groups consisting of care home experts (*n* = 10) primary care experts (*n* = 8) and hospice or specialist palliative care experts (*n* = 9). In each group, participants were asked to individually consider and note down what needs to be prioritised to support people to die well in each community setting by 2040 (home, care homes, hospice). A facilitator in each group guided participants through a structured process of i) brief discussion of critical issues emerging from the data; ii) individual recording of personal recommendations, and iii) sharing of individual recommendations with the wider group. A scribe wrote the recommendations on flipcharts, and group members agreed a prioritised order. Following group discussion, the top recommendations from each group were summarised by each group facilitator, typed and projected to the whole room. Finally, all participants identified individually their top three recommendations from those that emerged across all groups.

#### Data analysis

Individual recommendations, flipchart records of group priorities and final recommendations across all care settings were entered on Excel and categorised into themes. The top three recommendations, which at least 50% of participants prioritised during the final stage ordering, were identified. The individual and group-level priorities were examined to provide further detail relating to the top three recommendations that emerged.

### Ethics

For the trend analysis, we used anonymised, aggregate and publicly available routine data, which did not require ethical approval. The Usher Research Ethics Group, University of Edinburgh approved the expert consultation (No: 1862).

## Results

### Trend analysis

#### Recent mortality trends in Scotland

**Fig. 1:**
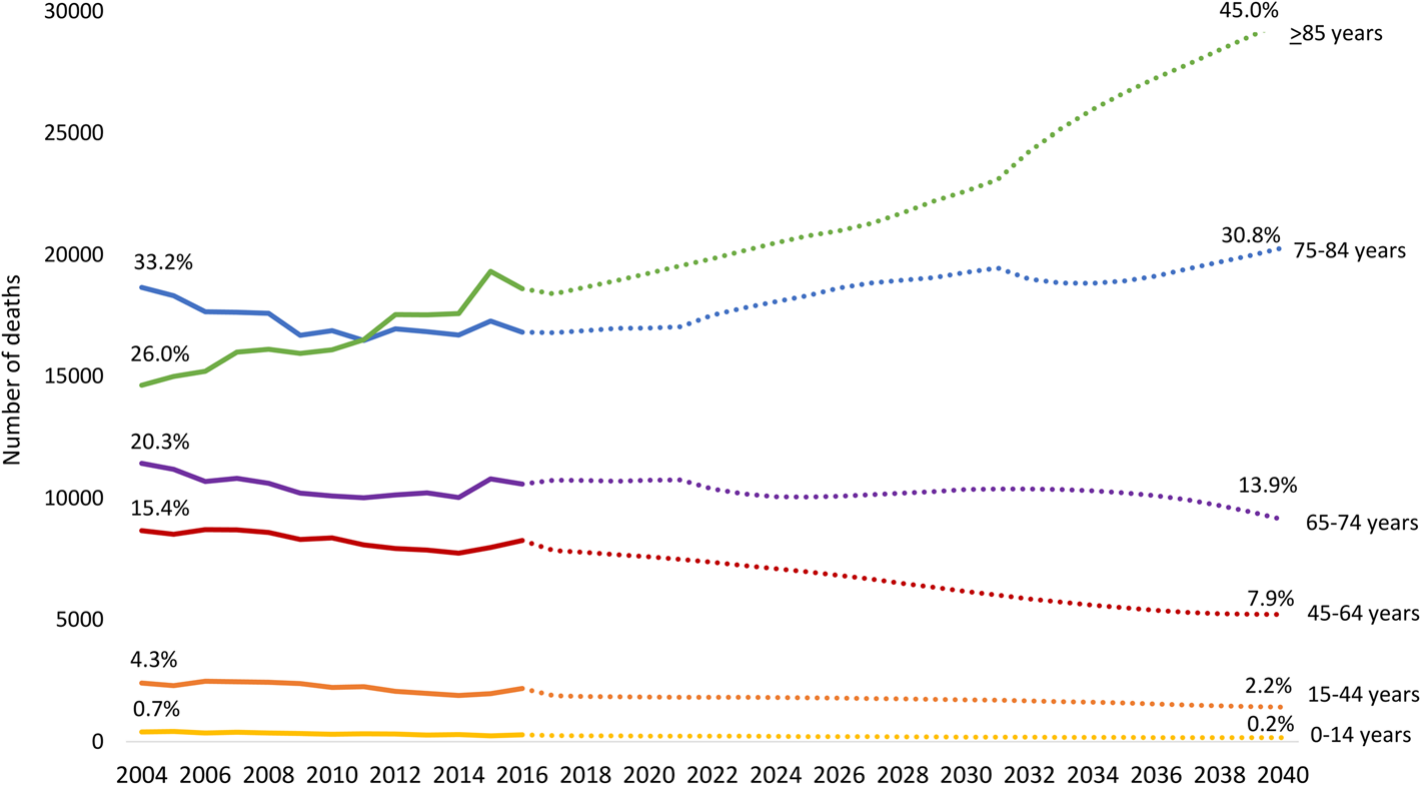
Number of past deaths (2004–2016) and projected future deaths in Scotland by age group (2017–2040)

Between 2004 and 2016, there was a mean of 55,260 deaths per year in Scotland. Deaths of people aged 85 or over increased from 14,634 in 2004 to 18,603 in 2016, while the number of deaths in all other age categories declined over this period (Fig. 1).

#### Projected mortality trends in Scotland

**Fig. 2:**
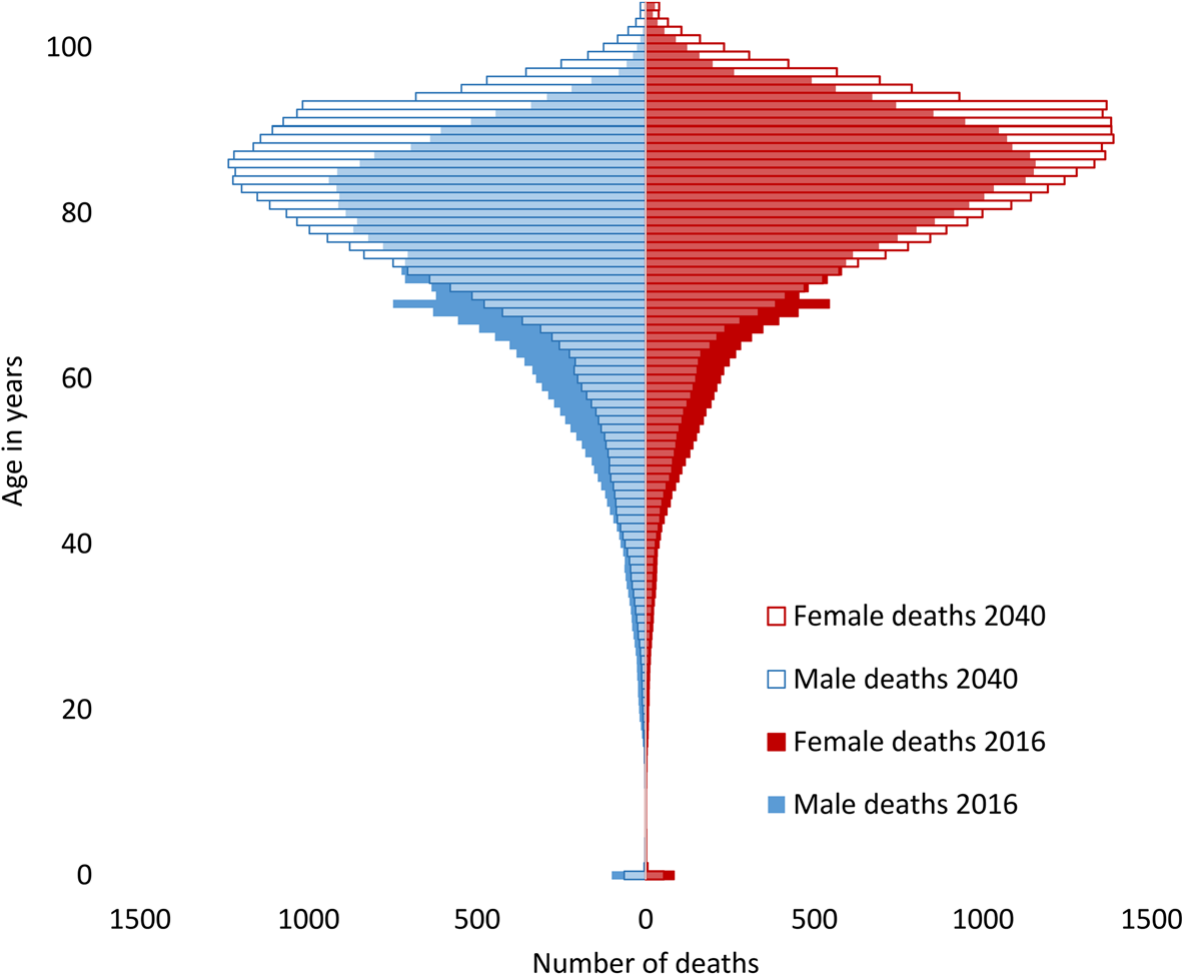
Deaths in Scotland in 2016 and projected future deaths in 2040 by age and gender

There are projected to be 65,757 deaths in Scotland in 2040, a 15.9% increase from 2016. Figure 2 shows deaths in 2016 and 2040 by age and gender, displaying a shift from younger to older ages, and a larger increase in male deaths. There are projected to be 10,960 more deaths (58.9% increase) of people age 85 years and over in 2040 compared to 2016. Deaths of people age 85 years and over accounted for 32.8% in 2016 and will account for 45.0% by 2040 (Fig. 1).

#### Recent trends in place of death in Scotland

**Fig. 3:**
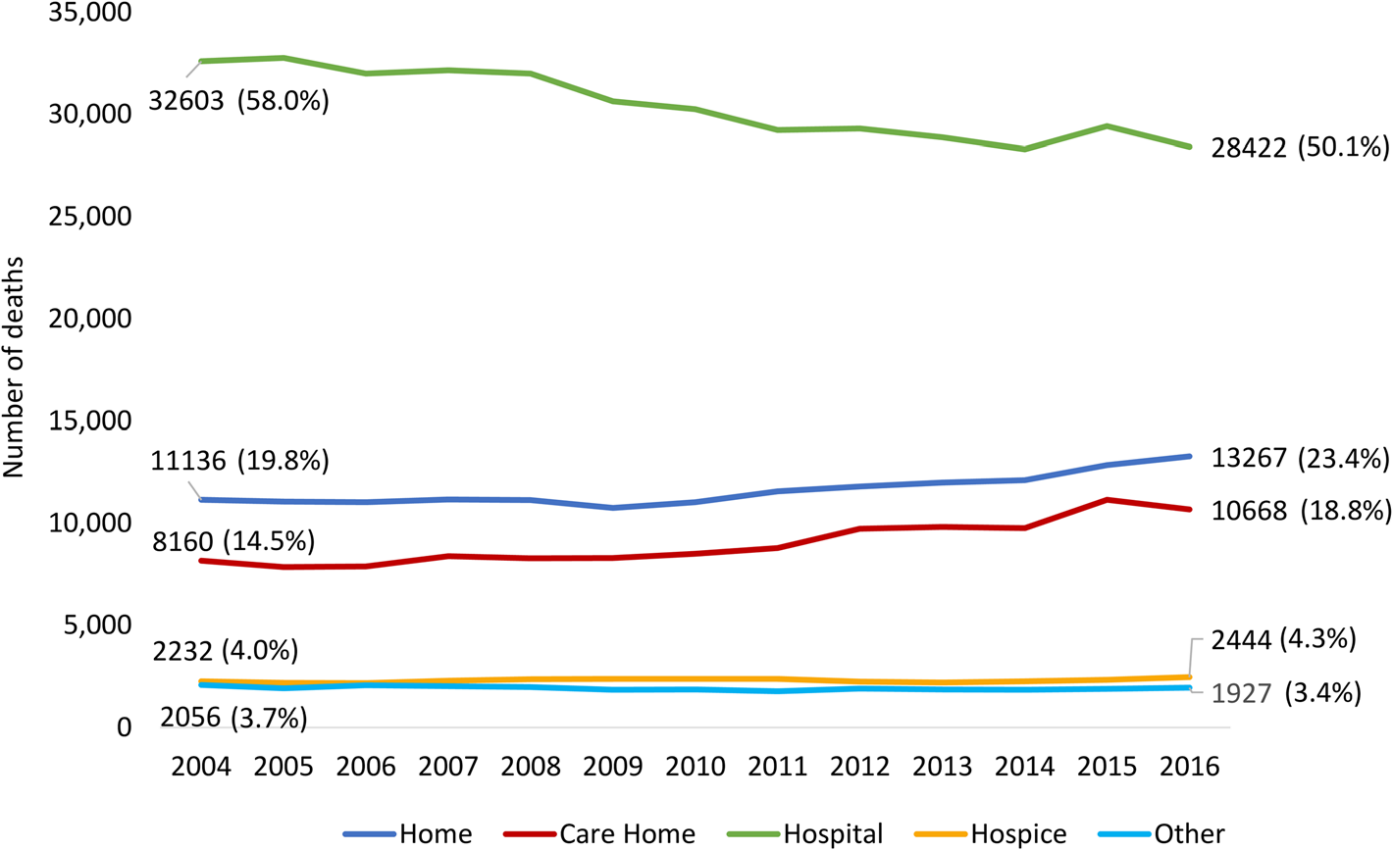
Number and proportion of deaths by care setting in Scotland (2004–2016)

In Scotland in 2016, most people died in hospital (*n* = 28,422, 50.1%), followed by home (*n* = 13,267, 23.4%) and care home (*n* = 10,668, 18.8%). A minority died in a hospice (*n* = 2444, 4.3%). Deaths at home, in a care home and hospice increased between 2004 and 2016 while deaths in hospital decreased (Fig. 3).

#### Projections of place of death

##### Scenario 1

**Fig. 4:**
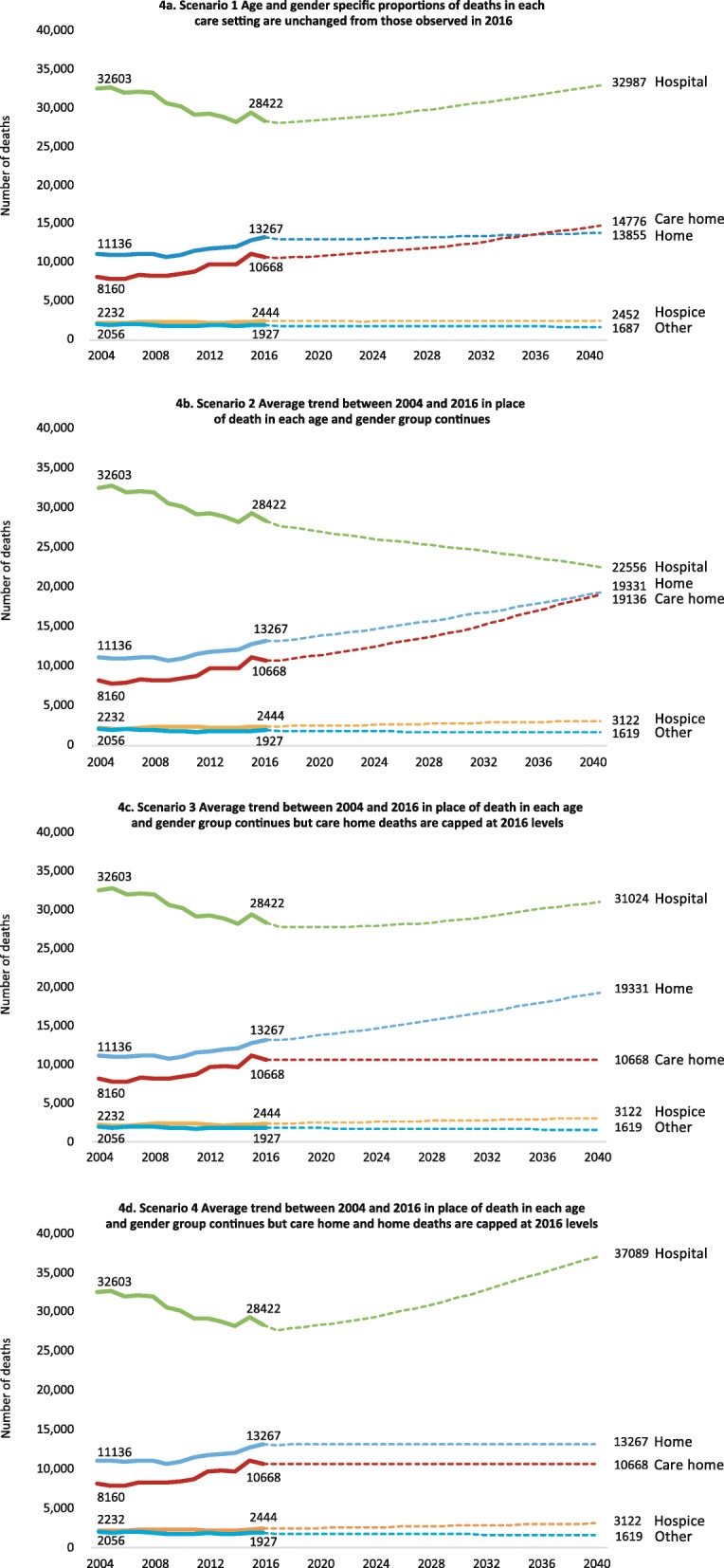
Past deaths (2004–2016) and modelled scenarios for future deaths in Scotland (2017–2040)

If age and gender specific proportions of deaths in each care setting remain unchanged from those observed in 2016, the number of deaths in hospital, home, care home, and hospice will increase (Fig. 4a and Table 1). Between 2016 and 2040, hospital deaths are projected to increase by 4565 deaths, but there will be little change in the proportion of people dying in hospital, which will remain the most common place to die (50.1% in 2016 and 50.2% in 2040). The annual number of home deaths is projected to increase by 588 deaths, representing a decline in the proportion of overall deaths (23.4 to 21.1%). Annual deaths in care homes are projected to increase by 4108 deaths, and increase as a proportion of all deaths (18.8 to 22.5%).

**Table 1:** Number and proportion of deaths in Scotland by place of death in 2004 and 2016 and projected estimates for 2040

	Observed deaths				Projected deaths in 2040							
	2004		2016		Scenario 1^a^		Scenario 2^b^		Scenario 3^c^		Scenario 4^d^	
	n	%	n	%	n	%	n	%	n	%	n	%
Home	11136	19.8	13267	23.4	13855	21.1	19331	29.4	19331	29.4	13267	20.2
Care home	8160	14.5	10668	18.8	14776	22.5	19136	29.1	10668	16.2	10668	16.2
Hospital	32603	58.0	28422	50.1	32987	50.2	22556	34.3	31024	47.2	37089	56.4
Hospice	2232	4.0	2444	4.3	2452	3.7	3122	4.7	3122	4.7	3122	4.7
Other	2056	3.7	1927	3.4	1687	2.6	1619	2.5	1619	2.5	1619	2.5
TOTAL	56187	100	56728	100	65757	100	65764	100	65764	100	65765	100

^a^Scenario 1: Age and gender specific proportions of deaths in each care setting are unchanged from those observed in 2016

^b^Scenario 2: Average trend between 2004 and 2016 in place of death in each age and gender group continues

^c^Scenario 3: Average trend between 2004 and 2016 in place of death in each age and gender group continues but care home deaths are capped at 2016 levels

^d^Scenario 4: Average trend between 2004 and 2016 in place of death in each age and gender group continues but care home and home deaths are capped at 2016 levels

##### Scenario 2

If the mean yearly changes in age and gender specific proportions of deaths in each care setting observed between 2004 and 2016 continue, we project that between 2016 and 2040, the annual number of people dying in hospital will decline by 5866 deaths, representing a decline in the overall proportion of deaths (50.1 to 34.3%). The number and proportion of deaths in community settings, including home, care home and hospice, is projected to increase. Between 2016 and 2040, the annual number of home deaths is projected to increase by 6064 deaths (23.4 to 29.4% of all deaths), care home deaths by 8468 deaths (18.8 to 29.1% of all deaths) and hospice deaths by 678 deaths (4.3 to 4.7%).

##### Scenario 3

If adding to scenario 2, **care home deaths do not increase** above the number observed in 2016, by 2040, we project that the annual number of hospital deaths will increase by 2602 deaths, and decline as a proportion of all deaths, from 28,422 to 31,024 (50.1 to 47.2%). The proportion of care home deaths, on the other hand, would decline from 18.8 to 16.2%, while the absolute number of care home deaths would remain around 10,668.

##### Scenario 4

If adding to scenario 3, **home deaths also do not increase** above the number observed in 2016, then hospital deaths will rise from 28,422 (50.1%) in 2016 to 37,089 (56.4%) in 2040. Proportionally, care home and home deaths would gradually decrease over the years, reaching 16.2% (10,668 deaths) and 20.2% (13,267 deaths) respectively, in 2040.

#### Transparent expert consultation

A transparent expert consultation was convened in October 2018 in Edinburgh. Twenty-seven experts participated comprising policy-makers, clinicians, health service managers, social care workers, educators and senior academics. The workshop generated 168 individual priorities. These were distilled down to 36 priorities during group discussions, which after further discussion and synthesis were reduced to 10. Overlapping recommendations were amalgamated by A.M.F and A.E.B, resulting in a final set of seven priorities. Of these, consensus was greatest for three recommendations, listed below with at least half of all participants rating them as one of their top three priorities. All seven priorities with examples from individual participants are shown in Additional file 2.

Top 3 priorities:

1. Increase, equip and sustain a skilled health and social care workforce through recruitment to community posts, education, training and valuing of care work.
2. Build community capacity and resilience by providing information, practical and financial support for carers and fostering community engagement initiatives.
3. Stimulate a realistic debate on death and dying, residential care and individual choice given funding constraints.

##### Workforce

There was consensus that workforce issues need to be urgently prioritised. Participants noted the need to increase the number of district nurses, social care staff, and GPs to meet the needs of the growing number of people expected to die by 2040. Participants agreed that the care home workforce and social carers need to be better valued for the work they do, and this should be reflected in financial reward as well as opportunities for career progression. Participants emphasized the value of training and the need to ensure all new care home staff have palliative care training during induction. They also stressed that specialist palliative care providers could build palliative care capacity in care homes through collaborative education and training initiatives.

##### Community capacity and resilience

Participants emphasized the importance of making information, practical and financial support more widely available to informal carers of people with advanced illness and providing opportunities for education and training of carers where appropriate. They highlighted the importance of community engagement initiatives that harness the contribution of volunteers to support people to remain at home should they wish.

##### Realistic debate

Participants called for a realistic and open debate on death, dying, and bereavement in society, and honest communication around what is realistic as opposed to ideal.

## Discussion

Our projections show that if current Scottish trends continue (Scenario 2), the need for end-of-life care will rise over the next 20 years, particularly in home and care home settings. By 2040 community settings could feasibly account for nearly two-thirds of all deaths, and hospital could fall to approximately one-third. These findings align with those projected in England and Wales [7]. However, if community support and capacity does not radically increase, these currents trends will not be sustained. If care home capacity remains at 2016 levels, hospital deaths could increase by 9.2% by 2040 (Scenario 3). If home deaths also remain at 2016 levels, hospital deaths could increase by 30.5%, representing 56.4% of all deaths by 2040 (Scenario 4). This means that most people would die in hospital, and at higher levels than was observed in 2004.

Our expert consultation findings support a continued shift toward more and better end-of-life care in the community. There is growing evidence to support the effectiveness of home based palliative care [14, 15]. Bainbridge et al. identify the critical components of effective home based palliative care, which include linkage with hospital and community services, a multidisciplinary team and holistic approach, and access to end-of-life care training and expertise [15]. These components were also prioritised by our experts, who identified collaboration and partnership working amongst their priorities (Additional file 2). Integrated models of care, such as those providing hospital to home services, or palliative care integrated with existing services also show promise and should be considered further [16–18].

If current trends continue (Scenario 2), 29% of people will die in care homes by 2040; this is nearly 80% more people than died in care homes in 2016 (10,668 to a projected 19,136). In Scotland, the number of care homes for older people decreased by 10% from 949 in 2007 to 854 in 2017, and the number of care home places fell, albeit at a slower rate (from 37,540 to 37,278) [19]. A reversal in this trend is needed if we are to support more people to die in the community. In Switzerland and the Netherlands, over a third of people die in care homes [20] and in Norway 45.5% of people died in a care home in 2011 [21]. In the Netherlands, there are ‘elderly care physicians’ specialised in care for the elderly who contribute to high quality care in care home settings [22]. In Norway, the shift from hospital to care homes deaths (observed since 1987) is attributed in part to changes in cause of death (from circulatory disease to cancer to dementia) alongside policies to shift care from hospital to care homes where appropriate [21]. With supportive policies and adequate funding, shifts in place of death are therefore possible.

Experts participating in our consultation prioritised the need to increase, upskill and sustain the community-based health and social care workforce to enable more people to die in a community setting. An increase in recruitment to primary care and community nursing is essential and is planned [23]. Critically, the social care workforce needs to increase despite significant challenges regarding recruitment to social care roles due to low pay, unsocial working hours and the emotional demands of care work [24]. Training in effective communication is essential [25] and there are approaches that could be used more widely to enhance skills [26–28], although evidence about ensuring sustained changes in practice is lacking [29]. Palliative care needs to be part of the curriculum for health and social care professionals, and students would benefit from opportunities to experience care of people with advanced progressive illness in primary care and care homes as part of their training [30]. This would need to be supported by appropriate tools to aid assessment and management, such as the Integrated Palliative Care Outcome Scale for Dementia (IPOS-Dem) [31, 32] and decision support [33]. Palliative care delivered in the community out-of-hours poses challenges to patient safety as most resources go into in-hours care [34]. Confidence in assessing care emergencies out-of-hours is lacking; education using flexible approaches such as e-learning are required to support GPs to deliver good emergency care at the end-of-life [35]. The social care workforce needs greater opportunities for training and development, with opportunities to progress within their role [36]. Hospices could better support community-based staff, particularly by delivering education and training in care homes and to the social care workforce. Ehealth approaches show promise and the use of video-conferencing to facilitate education and training across teams based in a variety of community settings is feasible, though further evidence of effectiveness is required [37, 38].

Experts highlighted the need to build community capacity and resilience through informal carer support and community engagement in end-of-life initiatives. Many people express a preference to die at home provided they get sufficient support and care, including home palliative care to feel safe and secure [5, 39–43]. Improving information, practical and financial support for both patients and informal carers is essential. There is a substantial network of informal carers involved in providing end-of-life care, many of whom are invisible to the health care team [44, 45]. Routine identification of informal carers is therefore needed to ensure that carers receive adequate support in their role [46]. The Carer Support Needs Assessment Tool (CSNAT) provides a formal approach to facilitate discussions with carers about their support needs [47], with evidence to support its effectiveness [48]. Carers may also benefit from training or education regarding practical nursing tasks and administering medicines, in person or using online resources [49–51]. Public health approaches that promote community engagement provide vital support outside of professional care [52, 53]. This can involve the development of supportive communities of volunteers who provide practical and/or emotional support to people at the end-of-life and their families. Innovative models of care involving community participation need to be developed and evaluated to support a shift in end-of-life care from acute to community settings.

Experts identified the need for a realistic debate on death, dying, residential care and individual choice in context of available funding. For many people, care home is the least preferred place to die [54], yet if current trends continue it is projected that more people will die in care home settings. There is a need to stimulate open discussion on quality and funding of end-of-life care in this challenging setting, so that everyone can expect a good standard of end-of-life care and can play a role in determining where they die. This recommendation is aligned with the concept of Realistic Medicine which has emerged in Scotland [55, 56], and promotes realistic and honest conversations about care, including dying and bereavement, putting the person receiving health and social care at the centre of decisions made about their care.

### Strengths and limitations

Our projections are not deterministic forecasts, rather scenarios based on different assumptions that can be used to inform decision-making regarding future resource allocation under certain circumstances. We assume a linear change over time, reflecting previous trends, however sudden changes in capacity (e.g. hospital closure) would result in a step-change in trends. The scenarios serve to stimulate debate, nationally and internationally, on what trends might occur under different conditions, and what trends may be best for people in Scotland.

A strength of our study is that we examine place of death for a whole country population in four key settings – hospital, home, care home and hospice. This allows comparison with other international studies and provides more nuanced data for service managers working in each and across all settings. However, these data do not shed light on patient transitions between settings and do not indicate time spent in the community versus hospital in the period before death. It also does not account for the type and quality of the care received in each setting. For example, hospice community palliative care clinical nurse specialists support many people to remain at home towards the end-of-life, yet the focus on place of death does not reflect this. Other scenarios are possible, e.g. fixed hospital capacity; and could be usefully considered alongside an economic analysis of each scenario in future studies.

Finally, a key strength is the incorporation of knowledge exchange in the study design. Engaging with a range of key stakeholders ensured that the interpretation of trend analysis findings was well-grounded in the context of health and social care provision in Scotland, with direct implications for practice and policy.

## Conclusion

If current trends continue, the numbers of deaths at home and in care homes will increase and two-thirds will die outside of hospital by 2040. However, this is very unlikely without additional investment in community-based care including care home capacity. Various actions are needed to maintain the trend: increasing and upskilling the community palliative care workforce; improving support for informal carers and encouraging community engagement in end-of-life care initiatives; and stimulating a realistic debate on death, dying and bereavement in context of funding constraints.

## Supplementary information

**Additional file 1.** Place of death data for Scotland: 2004–2016. Place of death sourced from the National Records of Scotland.

**Additional file 2.** Data from the Transparent Expert Consultation. List of the priorities identified with examples from individual participants.

## Data Availability

Place of death data analysed during the current study is available from the National Records of Scotland website: https://www.nrscotland.gov.uk/statistics-and-data/statistics/statistics-by-theme/vital-events/deaths. We categorised place of death according to NRS reporting standards, with the exception of hospice deaths, which were separated from the ‘care home’ category by the NRS at our request (Additional file 1). Data on projected deaths is available on the Office for National Statistics website: https://www.ons.gov.uk/peoplepopulationandcommunity/populationandmigration/populationprojections/compendium/nationalpopulationprojections/2016basedprojections

## Abbreviations

CSNAT: Carer Support Needs Assessment Tool
IPOS-Dem: Integrated Palliative Care Outcome Scale for Dementia
NRS: National Records of Scotland
ONS: Office of National Statistics
